# Prediction of Body Mass of Dairy Cattle Using Machine Learning Algorithms Applied to Morphological Characteristics

**DOI:** 10.3390/ani15071054

**Published:** 2025-04-05

**Authors:** Franck Morais de Oliveira, Patrícia Ferreira Ponciano Ferraz, Gabriel Araújo e Silva Ferraz, Marcos Neves Pereira, Matteo Barbari, Giuseppe Rossi

**Affiliations:** 1 Department of Agricultural Engineering, School of Engineering, Federal University of Lavras (UFLA), Lavras 37203-202, Brazil; franck.oliveira1@estudante.ufla.br (F.M.d.O.); gabriel.ferraz@ufla.br (G.A.e.S.F.); 2 Department of Animal Science, Federal University of Lavras (UFLA), Lavras 37203-202, Brazil; mpereira@ufla.br; 3 Department of Agriculture, Food, Environment and Forestry, University of Florence, Via San Bonaventura, 13-50145 Florence, Italy; matteo.barbari@unifi.it (M.B.); giuseppe.rossi@unifi.it (G.R.)

**Keywords:** digital livestock, precision livestock, dairy cattle, artificial intelligence, neural networks

## Abstract

The accurate prediction of body mass (BM) in cattle is crucial for herd monitoring, assessing biological efficiency, and optimizing nutritional management. This study evaluated BM prediction models using morphological data from 465 lactating Holstein cows, including the dorsal length (DL), thoracic width (TW), abdominal width (AW), rump width (RW), hip height (HH), body depth (BD), thoracic perimeter (TP), and abdominal perimeter (AP). Spearman’s correlation analysis identified TP (r = 0.89), AP (r = 0.88), and RW (r = 0.80) as the strongest predictors. Simple and multiple linear regression models, artificial neural networks (ANNs), and Support Vector Regression (SVR) were tested. The dataset was split into 90% for training (419 samples), 5% for validation (23 samples), and 5% for testing (23 samples). The best simple model, using only TP, achieved an R^2^ of 0.7763 and an RMSE of 43.69 kg. A multiple regression model with TP, AP, and RW improved performance (R^2^ = 0.9067, RMSE = 28.00 kg). The ANN outperformed all of the models (R^2^ = 0.9125, RMSE = 25.86 kg), and was followed by SVR (R^2^ = 0.9046, RMSE = 27.41 kg). As an indication of the evaluation of the results obtained, it is observed that, although regression models are effective, the ANNs and SVR provide greater accuracy, reinforcing their potential for herd management. However, simpler models remain viable alternatives for practical on-farm application.

## Simple Summary

Predicting body mass (BM) in dairy cattle is essential for efficient herd management, optimizing feeding strategies, and monitoring animal condition. Traditional methods, such as direct weighing, can be labor-intensive and impractical in large-scale production systems. This study explored the use of advanced computational techniques, including artificial neural networks (ANNs) and Support Vector Regression (SVR), alongside traditional regression models, to estimate the BM based on morphological data. Thoracic and abdominal perimeters were identified as highly correlated variables, enabling the development of high-accuracy predictive models. The findings highlight the potential of computational approaches to improve BM estimation, providing practical alternatives for the livestock sector. While more complex models demonstrated superior predictive performance, simpler statistical methods remain valuable options for on-farm adoption, balancing accuracy and ease of implementation.

## 1. Introduction

The measurement of body mass (BM) in dairy cattle can play a crucial role as a precision livestock farming tool by enabling precise and agile adjustments in nutritional management. BM data are essential for estimating maintenance nutritional requirements and dry matter intake [1] and can improve the accuracy of grouping dairy cows based on nutritional density requirements [2]. Monitoring BM changes around calving as a measure of energy balance can aid in animal health management, particularly in preventing disorders related to excessive body fat mobilization, which can lead to fatty liver and ketosis [3,4]. The adoption of precision nutrition practices can also enhance the diet formulation accuracy, positively impacting the economic performance and environmental footprint of dairy cows by reducing greenhouse gas emissions per unit of milk produced. Routine BM measurement per cow in commercial herds can also be a valuable tool for selecting smaller, biologically efficient animals by diluting the maintenance nutritional demand relative to lactation requirements [5]. Thus, accurate BM monitoring not only contributes to greater production efficiency but also aligns livestock farming with environmental and economic sustainability principles. This continuous BM monitoring allows producers to respond quickly to changes in the animals’ physical condition, maximizing livestock operation outcomes [6].

Traditionally, a ground scale is used to record the BM of cows. However, this practice is labor-intensive and stressful for both the animal and the operator [7,8], as the animal must be removed from its environment, guided to the weighing location, and immobilized, which can cause anxiety and discomfort, as well as pose a risk of accidents for the operator during handling. Scales placed at the exit of the milking parlor, at water trough entrances, or within voluntary milking system stalls have been used for the automatic weighing of lactating cows, but the availability of such equipment in herds remains limited in practice. Additionally, small-scale producers may not have the financial means to purchase commercial scales, as they can be expensive in some cases [6]. BM prediction can be performed using direct morphological measurements, such as the chest circumference, rump width, and hip width, which have been widely studied in the literature. Some authors [6,9,10,11] have demonstrated a significant positive correlation between these physical measurements and BM in cattle. Therefore, researchers have developed equations to relate biometric measurements to cattle body weight [6,12], highlighting the need for technologies capable of measuring BM in individual cows within herds that are cost-effective and easy to implement in practice.

It is evident that the intensification of livestock farming has brought significant changes, such as greater automation and specialization of processes, as well as the management of herds in more controlled and confined environments. Precision livestock farming, for instance, employs sensors and automated devices to collect real-time data [13]. Integrated digital precision livestock farming incorporates more advanced technologies, such as data analysis and artificial intelligence, enhancing these practices and promoting more efficient and sustainable herd management [14]. Machine learning algorithms have emerged as powerful tools in this context, enabling detailed analyses and accurate predictions that facilitate proactive management. In this regard, techniques like regression are widely employed to address tasks where the goal is to predict continuous values [15].

Artificial intelligence has revolutionized the prediction of cattle BM, providing more precise, efficient, and automated methods for livestock monitoring. The combination of traditional approaches, such as manual morphometric measurements [16,17,18], with modern techniques based on computer vision and machine learning [19,20,21,22] enables a more comprehensive analysis of the animals’ body condition. In this context, the development of algorithms for accurate BM prediction offers an efficient alternative for continuously assessing the physical condition of livestock. As technologies become more advanced and accessible, their implementation in commercial farms has become increasingly economically viable [22]. In addition to enhancing the accuracy of BM estimation, this integration facilitates more agile and informed management, optimizing productivity and sustainability in livestock farming. The reduction in the need for manual labor also contributes to minimizing animal stress levels and lowering operational costs associated with traditional approaches [23], making the adoption of these technologies even more advantageous. Furthermore, the adoption of these technologies enables the early detection of changes in the BM, allowing for swift and targeted corrective measures. This, in turn, improves the economic efficiency of livestock operations and supports more sustainable and responsible farming practices.

Although the use of machine learning for estimating cattle BM from morphometric measurements has gained attention, most previous studies rely only on simple linear regressions, without thoroughly exploring the potential of more complex algorithms. Additionally, there is a lack of research integrating detailed morphometric measurements with different machine learning techniques, which limits the evaluation of their performance in various scenarios. The literature also does not sufficiently explore the analysis of these algorithms considering a broader range of BM, which is essential to validate the robustness of the models under diverse conditions. This study addresses these gaps by utilizing more advanced machine learning methods and considering a wider range of BM, offering a relevant contribution to both the literature and practice, providing producers with a more efficient tool for herd management and monitoring. Therefore, this study aimed to estimate the BM of dairy cattle through the development and evaluation of machine learning models using physical dimensions of the dorsal and lateral regions of the animals, with the goal of providing a precise and efficient tool for herd monitoring and management.

## 2. Materials and Methods

### 2.1. Ethical Procedures

This research followed all experimental procedures approved by the Animal Ethics and Research Committee of the Federal University of Lavras, registered under CEUA No. 8093310125.

### 2.2. Data Collection

The study was conducted on a dairy cattle farm in the city of Ijaci, state of Minas Gerais, Brazil, at coordinates 21°09′40.1″ S 44°55′45.3″ W. Data were collected from lactating Holstein-Friesian cows, with 465 individual records used for analysis. The cows had a BM range of 420 kg to 855 kg and were housed in a tie-stall facility with sand bedding.

The cows were individually weighed using a Tru-Test digital scale, model EziWeigh5, with a resolution of 5 kg. Weighing sessions were conducted after the cows exited their first milking session of the day, which began at 5:00 a.m. and ended around 8:30 a.m. Upon leaving the milking parlor, the cows were handled and restrained in a chute, allowing for BM measurements in kilograms using the scale, as well as morphological measurements in centimeters using a measuring stick and a livestock measuring tape while the cows remained stationary at the site.

**Figure 1:**
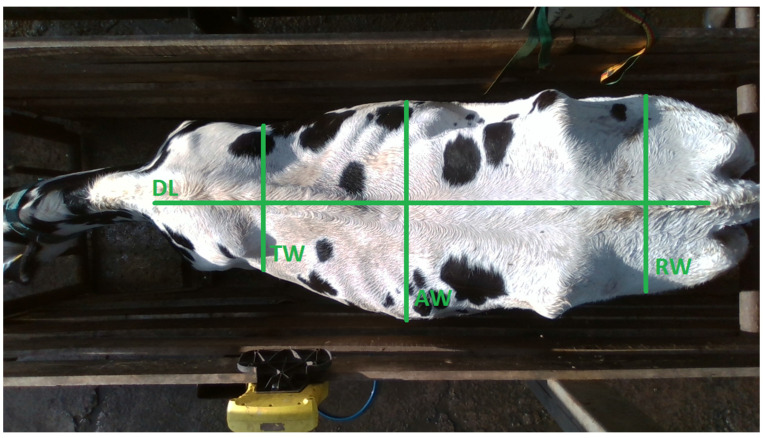
Dorsal measurements: DL—dorsal length; TW—thorax width; AW—abdomen width; RW—rump width.

In this study, along with the BM of the cattle, eight morphological measurements were collected, including four linear measurements of the dorsal region: dorsal length (DL), measured from the point where the neck meets the back to the base of the tail, at the point where the tail joins the body of the animal; thorax width (TW), measured between the most extreme points of the scapulae; abdomen width (AW), considered as the distance between the widest points in the abdominal region; and rump width (RW), defined as the widest point behind the cow’s hip bones [6,9,11,12,24,25,26]. These measurements can be visualized in Figure 1.

**Figure 2:**
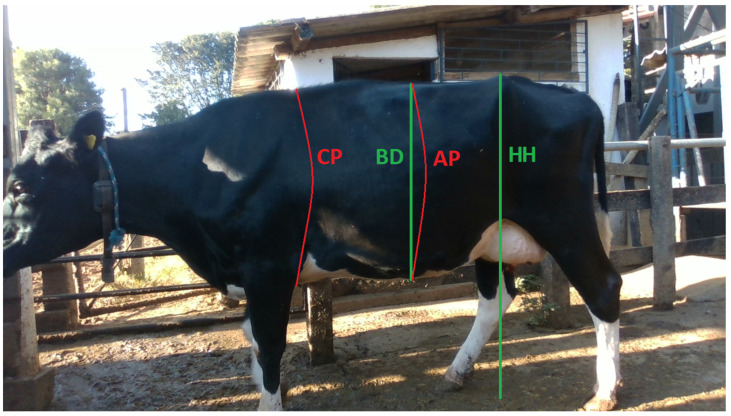
Lateral measurements and perimeters: HH—hip height; BD—body depth; CP—chest perimeter; AP—abdominal perimeter.

In addition to the dorsal measurements, two more linear measurements were taken on the lateral part of the cattle (Figure 2): hip height (HH), measured from the highest point of the hip bones to the ground [6,12,24,25,26,27], and body depth (BD), measured as the distance between the longest points of the abdomen in lateral view [26]. To complete the eight measurements, two perimeter measurements were also taken: chest perimeter (CP), also referred to in the literature as heart girth (HG) or chest girth (CG), measured around the circumference of the animal’s body in the thoracic region, just behind the forelimbs [6,9,12,24,27]; and abdominal perimeter (AP), measured around the circumference of the widest part of the abdomen.

**Figure 3:**
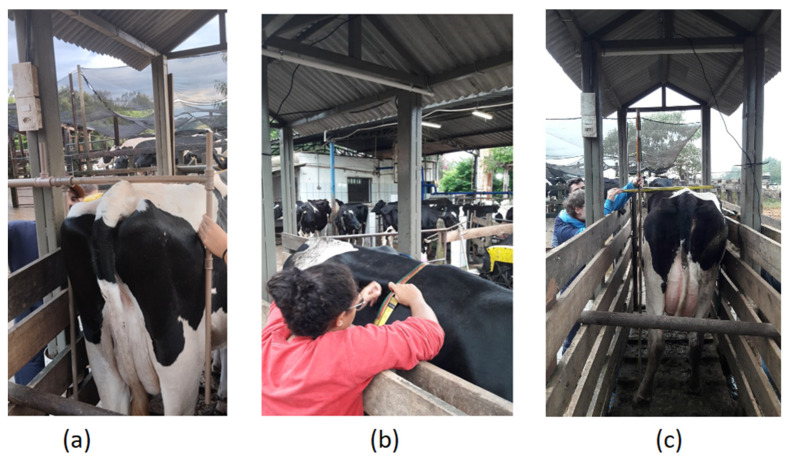
Data collection process. (**a**) measurement of RW (rump width) using a measuring stick; (**b**) measurement of AP (abdominal perimeter) using a cattle measuring tape; (**c**) measurement of HH (hip height) using a cattle height stick.

The measurements for data collection on a sampling day can be visualized in Figure 3.

### 2.3. Statistical Analysis

#### 2.3.1. Data Normality Analysis

Given the considerable number of observations, the Kolmogorov–Smirnov (KS) test [28,29] was employed to rigorously assess the normality of the variables. This test is suitable for large samples and is more appropriate than, for example, the Shapiro–Wilk test [30] in this context. It allows for a comparison between the data distribution and the expected normal distribution. Based on the results of the normality test, the choice between Pearson’s or Spearman’s correlation can be made on a solid basis, ensuring the validity of the conclusions.

#### 2.3.2. Data Correlation Analysis

Spearman’s correlation [31] was chosen to analyze the relationship between morphological variables and BM due to its suitability for data that do not require a normal distribution or a linear relationship between variables. This method was employed to identify the strength and direction of associations among the variables of interest, enabling a robust analysis of correlation patterns.

#### 2.3.3. Regression Analysis

Simple linear regression and multiple linear regression analyses were conducted using the statistical software RStudio (version 2024.04.2). In addition, normality and correlation analyses were also performed in this software to identify the morphological variables with the highest correlations with bovine BM, providing insights into the relationships between the data.

### 2.4. Development of ANN

The dense neural network was selected due to its ability to model complex and non-linear relationships between input variables and the target variable. The input variables were CP, AP, and TW, while the variable to be estimated was BM. The network development included defining the network architecture, tuning hyperparameters, and training with the specific dataset to ensure robust and accurate performance.

The neural network was developed using Google Colab, a cloud-based platform that provides advanced computational resources and an interactive environment for Python code development. Google Colab facilitates the use of deep learning libraries such as TensorFlow and Keras, offering a free and accessible environment with support for GPUs and TPUs, which significantly accelerates the model training process. Utilizing Google Colab, the neural network was designed based on the most significant morphological variables.

#### 2.4.1. Dense Neural Network (Multi-Layer Perceptron)

For the BM regression task, a Multi-Layer Perceptron (MLP) neural network with a tanh activation function was employed. The choice of this model was motivated by its ability to handle non-linear regression problems and the flexibility provided by its relatively simple architecture. This architecture includes an input layer, hidden layers with tanh activation, and a linear output layer [32].

The tanh activation function in the hidden layer transforms the input data, enabling the network to model non-linear relationships between morphological variables and the target variable. Tanh aids in normalizing activations and enhances convergence during training, making it an effective choice for fitting complex data and performing interpolation. To ensure the functionality of the neural network, the input data were normalized before training, and both input and output values were scaled to fall within the range [−1, 1] [33].

Moreover, dense neural networks using the tanh activation function provide the advantage of relatively fast training and strong generalization capabilities, enhancing the robustness of the model in predicting BM based on new data. This non-linear approach complements the analyses performed with simple and multiple linear regression models, offering a more comprehensive perspective on the predictive capacity of the methods used.

#### 2.4.2. Hyperparameters

Hyperparameters are model variables that control the behavior of the model and the overall architecture of the ANN. These are specified by the user prior to the training process and are, in most cases, static (they do not change during training) [34].

Depending on the problem at hand (regression or classification), it is necessary to define the appropriate loss function, such as Categorical Cross-Entropy for multi-class classification or Mean Squared Error (MSE) for regression, and choose among various optimization algorithms, such as Adam, RMSprop, or SGD. Considering the dataset size and the available computational resources, the maximum number of training epochs and the batch size for training are defined [34]. These choices have a direct impact on the model’s performance.

The dataset was split into 90% for training (419 samples), 5% for validation (23 samples), and 5% for testing (23 samples) for the regression model with the output being the predicted BM. The learning rate was set at 0.0001, and the performance of the MLP ANN was tested using various optimizers (Adam, SGD with momentum values of 0.85, 0.90, and 0.95, and RMSprop), batch sizes (8, 16, and 32), different numbers of hidden layers (2 and 3), varying numbers of neurons per layer, and a fixed number of 5000 epochs. The final MLP architecture for BM estimation was selected based on the highest *R*^2^ and the lowest RMSE and MAE for both training and testing datasets.

This approach ensured that model performance was continuously monitored during training, through backpropagation, which is the standard training method used by the Keras package, allowing for the identification of potential overfitting issues. In the case of the MLP, the validation set played a crucial role in selecting the best hyperparameter combination, helping to define the most suitable architecture before the final evaluation on the test set. Additionally, the exploration of multiple hyperparameters, such as optimizers, batch sizes, and different network architectures, provided further control over the learning process and contributed to a robust model selection. In this way, hyperparameter tuning was conducted based on performance metrics obtained directly from the validation set, ensuring that the network was trained with a good balance between bias and variance. Therefore, there was no need to use cross-validation or other more specific techniques to validate the data, as the adopted methodology already allowed for a reliable assessment of the model’s generalization. In the other regression models, the data split followed a logic consistent with the smaller number of hyperparameters involved in these algorithms, making it feasible to directly evaluate their performance without requiring intermediate validation.

### 2.5. Support Vector Regression (SVR)

Support Vector Regression (SVR) is an extension of the Support Vector Machine (SVM) algorithm [35,36] designed for regression tasks. While SVMs are primarily employed for classification, SVR is used to predict continuous values instead of discrete categories.

In this study, SVR was applied to predict the BM of cows based on morphological data, including the variables CP, AP, and RW. SVR is particularly effective for capturing complex relationships between variables, offering accurate predictions even in the presence of data variability and outliers.

## 3. Results and Discussion

**Figure 4:**
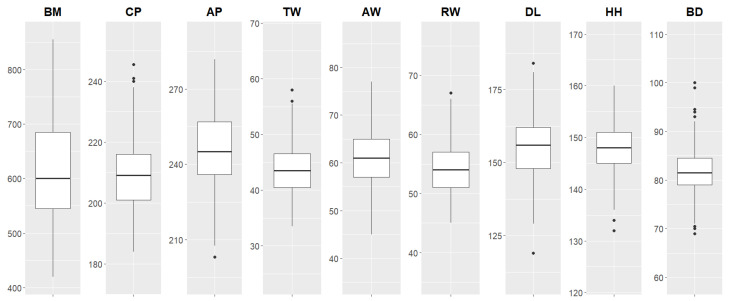
Box plot of the variables analyzed in centimeters: BM = body mass; CP = chest perimeter; AP = abdominal perimeter; TW = thorax width; AW = abdomen width; RW = rump width; DL = dorsal length; HH = hip height; and BD = body depth.

The descriptive analysis of the variables analyzed is presented in Figure 4 using a box plot.

The predictive models analyzed in this study are based on variables whose distributions are presented in Figure 4, showcasing descriptive statistics through boxplots. These models were developed considering the observed value ranges for each variable. The variation intervals highlighted in the boxplots represent the boundaries considered in the model analyses, directly reflecting data variability and its relationship with BM prediction.

The ranges of the variables measured in this study exhibit significant variations, reflecting the diversity of the animals observed, especially within the context of precision livestock farming. The BW of the animals in this study ranges from 420 to 855 kg, with an average of 612.96 kg, showing a broader range compared to other studies.

For instance, the authors of [24] reported an average BW of 513.4 kg for Holstein-Friesian cattle, while [9] found an average of 440.21 kg for the Ongole breed. Similarly, the authors of [26], in a study on Holstein-Friesian cattle, identified a range between 441.76 and 519.62 kg, with an average of 481.87 kg. Additionally, the authors of [6] analyzed the Girolando breed and found a range of 360 to 596 kg, with an average of 473 kg.

The greater variation range in the data presented here, despite being focused exclusively on the Holstein-Friesian breed, may be associated with factors such as different nutritional management conditions, lactation stages, or the age of the animals. These elements contribute to a comprehensive assessment of the herd in the context of precision livestock farming, enabling efficient adaptation of management practices and interventions according to the animals’ body condition.

It is possible to observe some trends in the averages of other variables analyzed when compared to results from other authors. The average DL in this study, at 155.25 cm, is higher than reported in [24] (146.37 cm) and [9] (143.20 cm), reflecting morphological diversity among the animals. On the other hand, the HH value of 147.98 cm in this study shows consistency, though slightly higher, compared to the averages reported in [24] (137.20 cm) and [26] (136 cm), indicating a certain uniformity in this specific variable. Meanwhile, CP, with a higher average (209.43 cm) compared to that reported in other studies, such as [24] (189.36 cm) and [9] (175.18 cm), reinforces the hypothesis of a greater variability in body dimensions, which may directly impact the efficiency of predictive models for BM.

The analysis of the morphological ranges observed in the studied herd underscores the importance of considering the specific characteristics of the animal group when developing predictive models. The variation range in body traits can significantly influence the performance of algorithms, as such models tend to better adapt to the specific conditions of each herd. In this study, the herd presented larger dimensions compared to those reported by other authors in the literature, which may impact the model results and highlight the need for customization to reflect local characteristics. These differences emphasize the importance of tailoring models to the specific farm conditions, ensuring greater accuracy in BM estimation and optimizing management in precision livestock farming.

### 3.1. Results of the Data Normality Analysis

The results of the KS test are presented in Table 1; it was performed to verify the adherence of the distributions of the morphological variables to a normal distribution.

The results of the normality analysis using the KS test revealed that variables such as CP, AP, AW, DL, and HH exhibit distributions that can be considered normal, based on the obtained *p*-values (*p* > 0.05) [28]. This observation aligns with the rigorous statistical analysis provided by the KS test. On the other hand, other variables, including BM and RW, did not show significant adherence to the normal distribution (*p* < 0.05).

Given these results, the Spearman correlation coefficient was used for all correlation analyses involving BM, as it is the target variable, regardless of the distribution of the other variable. This ensures robustness in the face of BM’s non-normality and captures monotonic associations between the variables [37].

### 3.2. Results of Data Correlation Analysis

**Figure 5:**
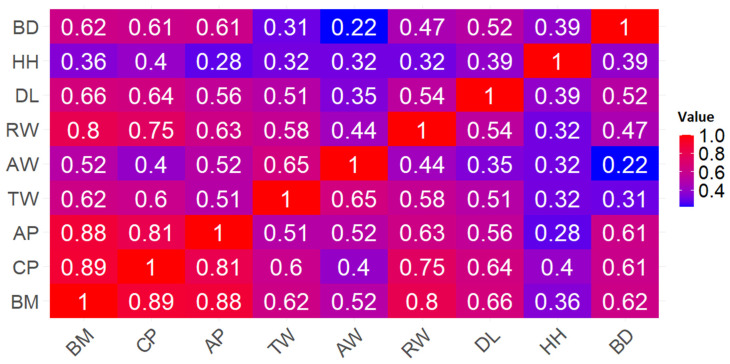
Correlation matrix between variables: BM = body mass; CP = chest perimeter; AP = abdominal perimeter; TW = thorax width; AW = abdomen width; RW = rump width; DL = dorsal length; HH = hip height; and BD = body depth.

The Spearman correlation analysis was conducted to examine the relationship between BM and the selected morphological variables. Figure 5 illustrates the Spearman correlation coefficients for each pair of variables, highlighting the significant associations and the strength of these relationships.

The three variables with the highest individual correlations with BM were CP (r = 0.8948), AP (r = 0.8805), and RW (r = 0.8020). Similarly, the study conducted by the authors of [7] extracted morphological measurements from digital images obtained using a Microsoft Kinect device and found significant correlations between body measurements and the BM or carcass weight of beef cattle. In that study, the chest width (thoracic width) was the variable most closely related to weight, with correlations exceeding 0.85, highlighting the potential of these measurements for estimating body weight. However, it is worth noting that the referenced study used a smaller sample size (35 samples) compared to the present study. Therefore, the results of this study may provide a slightly more realistic and robust understanding of the correlations between morphological variables and BM due to the larger dataset analyzed.

According to [38], correlation coefficient values between 0.7 and 0.9 are considered high positive correlations. Based on these values, the three morphological variables (CP, AP, and RW) with correlation coefficients within this range were selected to evaluate and compare subsequent algorithms. This criterion was adopted to ensure that the selected variables have a strong and significant relationship with BM, increasing the reliability of subsequent analyses.

On the other hand, correlation coefficient values between 0.5 and 0.7 are considered moderate correlations [38]. These variables were not included in the main analyses, as the focus is on stronger relationships capable of providing clearer and more precise insights into the influence of morphological variables on BM.

### 3.3. Linear Regression Results

**Figure 6:**
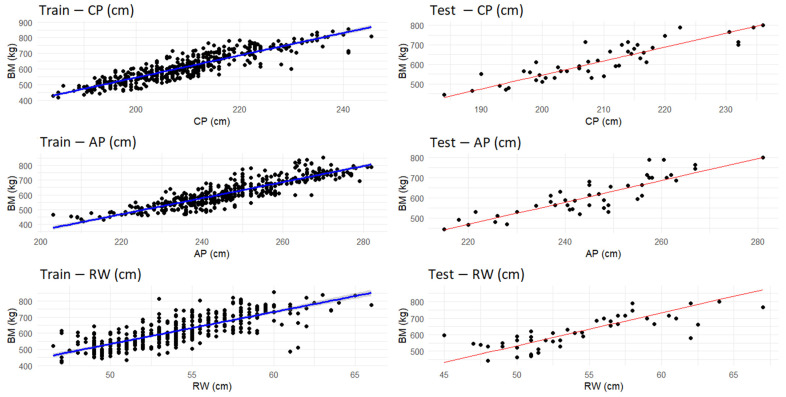
Training and testing graphs of simple linear regressions of significant variables.

Considering the significant variables identified by the Spearman correlation (CP, AP, and RW) (Figure 6), individual regression models were developed. These models aim to explore the relationship between the morphological variables and BM of the cows, assuming a linear approach to investigate how each independent variable contributes to the variation in BM. The data were randomly divided into training (90%) and testing (10%) sets to evaluate the performance of the models in predicting BM.

The training and testing graphs for the models can be seen in Figure 6.

The visual fit of the regression line to the training data demonstrates a robust model performance, as the points remain close to the trend line, reflecting prediction accuracy. This behavior is also observed in the test data, suggesting that the model generalizes well and minimizes the risk of overfitting. Despite a considerable variability in BM ranging from 420 kg to 855 kg, the proximity of the points to the line, both in training and testing, reinforces the achieved fit. These results are supported by the values presented in Table 2, where the coefficient of determination R^2^ reflects the model’s ability to explain most of the variability in the data. This graphical and numerical consistency demonstrates the adequacy of simple linear models to capture the relationship between predictor variables and BM, despite the wide variation among individuals. Table 2 displays the training and testing performance results for these models.

The CP model demonstrates a good fit for both training and testing data, with R^2^ values of approximately 0.80 and 0.78, respectively. This suggests that about 78% of the variability in BM can be explained by CP. The relatively small difference between the R^2^ values for training and testing indicates that the model generalizes well to new data. The MAE and RMSE values show that the average prediction errors are reasonable, although there is room for improvement. Equation 1 represents the BM prediction model using the CP variable, with standard deviations for each term shown in parentheses.

BM_CP_ (kg) = −879.05 ( ± 37.019) + 7.1235 ( ± 0.176) × CP (1)

These results are comparable, though slightly inferior, to those obtained by the authors of [39], who used CP as a predictor variable in a study involving 38 Holstein cows and achieved an R^2^ of 0.89 (*p* < 0.01). The difference in R^2^ values can be attributed to factors such as sample size and the specific characteristics of the study populations. While the present study shows lower accuracy, it reinforces the effectiveness of CP as a relevant predictor variable for bovine BM.

The AP model also demonstrates a solid performance, with a training R^2^ of 0.7682 and a testing R^2^ of 0.7656. This indicates that approximately 77% of the variability in BM can be explained by AP. However, the MAE and RMSE values are higher than those of the BM_CP_ model, suggesting that the predictions are slightly less accurate. Equation 2 corresponds to the BM prediction model using the AP variable.

BM_AP_ (kg) = −728.29 ( ± 36.048) + 5.443 ( ± 0.146) × AP (2)

The RW model exhibits a weaker performance compared to that of the BM_CP_ and BM_AP_ models, with a training R^2^ of 0.6062 and a testing R^2^ of only 0.5484. This indicates that only about 55% of the variability in BM can be explained by RW. The higher MAE and RMSE values reveal that the predictions are significantly less accurate, with larger average errors. Equation (3) corresponds to the BM prediction model using the RW variable.

BM_RW_ (kg) = −464.35 ( ± 41.244) + 19.948 ( ± 0.762) × RW (3)

The results suggest that simple regression models can explain a substantial portion of the variability in BM, especially the CP and AP models. However, these models rely on only one independent variable, which may limit their ability to capture the full complexity of the relationships between morphological variables and BM.

The use of multiple regression could potentially improve the model’s fit. By simultaneously including multiple independent variables, it becomes possible to capture interactions and combined effects that are not evident in simple models [40]. This approach could result in a higher adjusted R^2^, indicating better explanatory power, as well as lower MAE and RMSE values, reflecting more accurate predictions.

The significant difference between the high Spearman correlation values and the results of simple regression models can be attributed to the nature of the methods. Spearman’s correlation measures the monotonic association between two variables [31], whereas simple linear regression assumes an exact linear relationship between the variables [41]. Spearman’s correlation is less sensitive to outliers and does not account for residual variability [37], which may explain its higher values compared to simple regression models.

### 3.4. Multiple Linear Regression Results

Multiple linear regression was employed to quantify the relationship between the independent morphological variables (CP, AP, TW, AW, RW, DL, HH, and BD) and BM. This approach allows for assessing the combined impact of physical characteristics on BM prediction, providing a more comprehensive understanding of the interactions among the variables.

In the first multiple regression model, only the three variables that showed the highest correlation with BM, according to the Spearman analysis—CP, AP, and RW—were used. The data were divided, with 90% for training and 10% for testing the model. Table 3 summarizes the estimated coefficients for each predictor variable using the training data, along with the associated *p*-values, indicating the statistical significance level of each coefficient concerning the BM of the cows studied.

Table 4 shows the performance metrics obtained for this model.

This model showed a strong performance, with an R^2^ of 0.8959 in the training set and an R^2^ of 0.9067 in the test set, indicating a strong ability to explain the variability of BM using the selected variables. The performance metrics, with an MAE of 22.29 and RMSE of 28.00, further reinforce the model’s effectiveness in predicting BM with high accuracy.

Therefore, the multiple regression equation (Equation 4) considering these three predictors is formulated as follows, with the standard deviation of each term in parentheses:

BM_M1_ (kg) = −988.7353 ( ± 27.000) + 2.8999 ( ± 0.247) × CP + 2.6445 ( ± 0.170) × AP + 6.3471 ( ± 0.580) × RW (4)

where −988.7353 is the intercept and in parentheses are the standard deviations of each term.

These results suggest that the three selected variables are good predictors of BM, providing a solid foundation for predictive models. In this context, the authors of [9] conducted a study to estimate the BM of Ongole cattle using CP and DL measurements, achieving a high coefficient of determination (R^2^ = 0.97) in predicting BM. The multiple regression analysis conducted by the authors revealed that CP and DL were fundamental for the model. The high accuracy may be associated with a division of cattle into age groups, ranging from 2.5 to 7.5 years. This segmentation may have promoted greater homogeneity within each age class, which in turn may have improved the model’s accuracy. The inclusion of different techniques and the consideration of data characteristics, such as age segmentation, highlight the importance of adjusting modeling methods to maximize accuracy in estimating cattle BM.

However, to explore whether including other variables could further improve the accuracy and robustness of the model, a new analysis was conducted considering all available variables. The model incorporated all the measured morphological variables. The analysis was performed using the same multiple linear regression method, maintaining the data split of 90% for training and 10% for testing.

Table 5 and Table 6 present the results of this comprehensive model, comparing its performance metrics with the initial model to assess the potential gains in predictive capability.

The results showed that this model exhibited a slightly superior performance in terms of the R^2^ in the training set (0.8997) compared to the first model (Equation (4)), which only used the three variables with the highest correlation. However, the R^2^ in the test set (0.9063) was slightly lower than that of the initial model.

The performance metrics of Model Multiple 2, with an MAE of 22.20 and an RMSE of 28.05, are very similar to those of Model Multiple 1. Although the R^2^ of Model Multiple 2 is slightly higher in the training set, the differences in error metrics are minimal, indicating that the addition of additional variables did not provide a substantial improvement in the model accuracy.

These results suggest that, although the model using all variables can capture more variability in the training data, it does not necessarily translate into a significant improvement in the predictive capability on new data. This may indicate that the three initial variables (CP, AP, and RW) already capture most of the relevant variability for BM prediction.

Furthermore, the similarity in error metrics between the two models reinforces the idea that adding variables with lower correlation does not contribute enough information to justify the added complexity of the model. This phenomenon may be due to redundancy or multicollinearity [42,43] among the additional morphological variables, which do not independently contribute to the improvement of predictive accuracy.

In conclusion, while Model Multiple 2, which includes all variables, shows a slight gain in terms of R^2^ in the training set, the performance metrics suggest that Model Multiple 1, with the three most correlated variables, is already sufficiently robust and effective. These results highlight the importance of selecting predictor variables based on their correlation and relevance, avoiding the inclusion of excessive variables that may not add significant value to the model.

It is also possible to state that multiple regression analysis allows for the exploration of interactions between variables that are not captured by simple correlation analysis. While in the correlation matrix, each variable is evaluated independently in relation to the dependent variable, multiple regression considers how independent variables interact with each other to explain variations in the dependent variable. This approach provides additional insights into how specific combinations of variables can influence the cows’ BM, emphasizing the importance of considering not only individual correlations but also interactions between morphological attributes.

Therefore, Equation (5) of multiple regression considering all the measured variables is formulated as follows:

BM_M2_ (kg) = − 926.2092 ( ± 48.559) + 2.7509 ( ± 0.291) × CP + 2.3619 ( ± 0.197) × AP + 0.8203 ( ± 0.532) × TW + 0.6406 ( ± 0.365) × AW + 5.6785 ( ± 0.594) × RW + 0.3929 ( ± 0.203) × DL − 0.8360 ( ± 0.382) × HH + 0.7616 ( ± 0.411) × BD (5)

where −926.2092 is the intercept and in parentheses are the standard deviations of each term.

**Figure 7:**
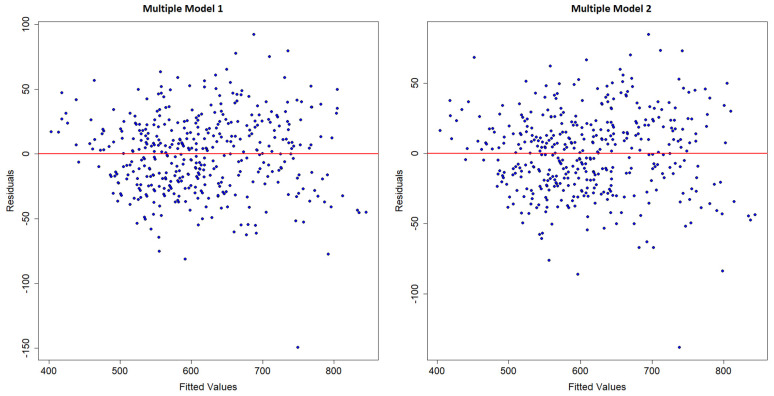
Relationship of residuals with adjusted values.

In addition to the analyses already performed with the test data, including the evaluation of the adjusted R^2^, RMSE, and MAE, the residuals were used to evaluate the multiple models created. The residuals vs. adjusted values plots are presented in Figure 7.

The plot of residuals versus fitted values reveals a scattered distribution of residuals around zero, with most points ranging between −50 and +50. This indicates that the fitted model does not exhibit systematic trends in the residuals, suggesting that the prediction errors are random and, therefore, the model is adequate. However, the presence of some points with residuals below −50 and a few close to −100, as well as a notable outlier with a residual of approximately −150, indicates significantly larger prediction errors for certain specific observations. These points are considered outliers and may warrant further investigation to understand the reasons for these discrepancies. Overall, the distribution of residuals supports the assumption of homoscedasticity (i.e., constant variance of errors), which is a positive aspect for the validity of the multiple linear regression model.

### 3.5. Neural Network Results

**Figure 8:**
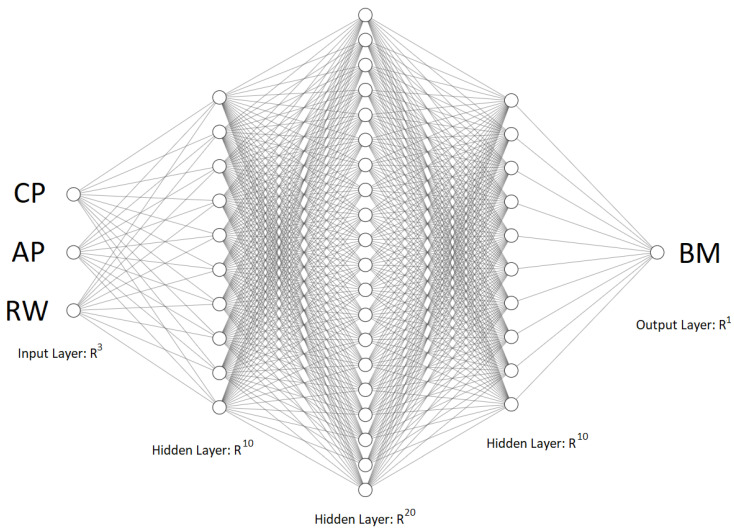
Final model.

In this section, the results obtained by using the MLP Network for the regression of the dependent variable BM of the cows, based on the independent variables CP, AP, and RW, are presented. Table 7 shows the hyperparameters selected for the neural network and the final model considered can be seen in Figure 8.

**Figure 9:**
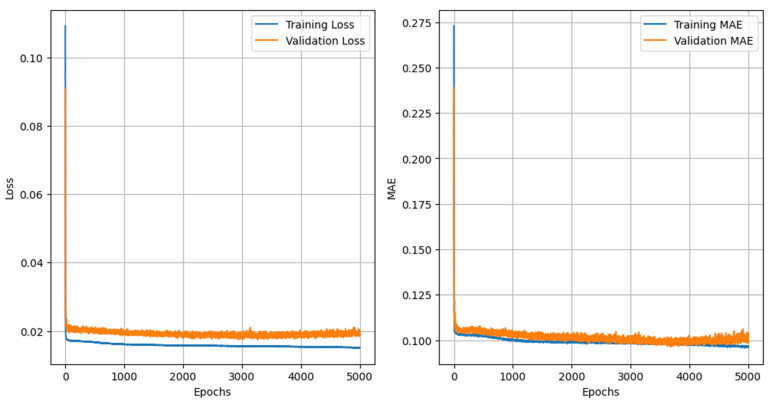
Training loss and MAE graph.

The loss and MAE graphs are presented in Figure 9 to illustrate the behavior of the data during training.

**Figure 10:**
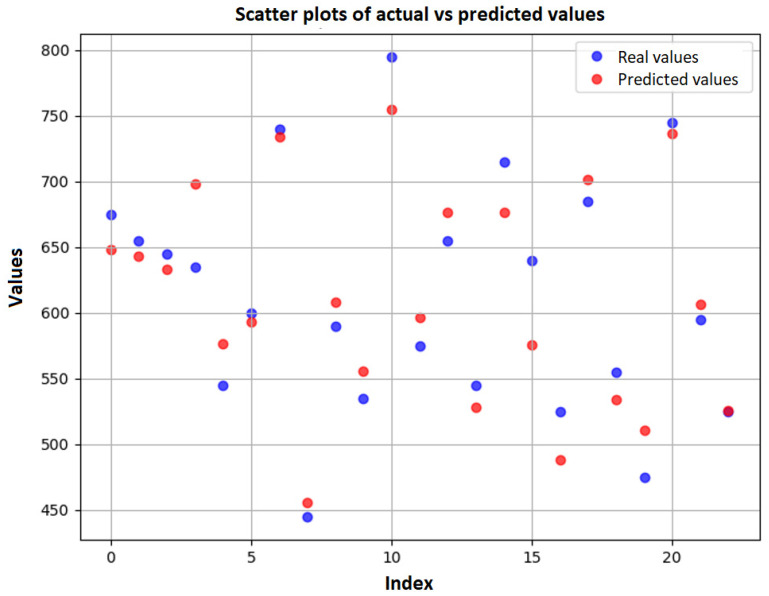
Visualization of observed and predicted values with test data.

Finally, a scatter plot is presented (Figure 10) to illustrate the relationship between the actual values and the values estimated by the neural network model, allowing for a clear visualization of the accuracy of the predictions.

To ensure better visualization and comparison of the results, Table 8 presents a summary containing the key performance metrics of the MLP model.

When analyzing the scatter plot, it is possible to observe that, despite the high correlation between the actual values and the values estimated by the neural network model, there is still a small prediction error. This error is evidenced by the R^2^ values in the training set (0.9139) and in the test set (0.9125), suggesting that the model has a good generalization capacity. However, the MAE (21.97) and RMSE (25.86) values indicate that, although the predictions are close to the actual values, there is still a deviation that can be attributed to variations in the data or limitations of the model in capturing all the nuances of the input data.

### 3.6. Support Vector Regression (SVR) Results

The results obtained for the SVR can be seen in Table 9.

The results obtained for the SVR model using morphological variables (CP, AP, and RW) demonstrate a good fit for both the training and test sets. Studies such as [44] show that SVR models with specific body measurements, such as length and circumference, are also effective in predicting the cattle carcass weight, even with differences in breed and rearing conditions. However, exploring other configurations, such as combining image or volume variables, could potentially further improve the model’s accuracy and applicability. This demonstrates that SVR, even in its current configuration, has great potential to be adapted to different zootechnical scenarios, contributing to more precise estimates of specific cattle parts or BM.

**Figure 11:**
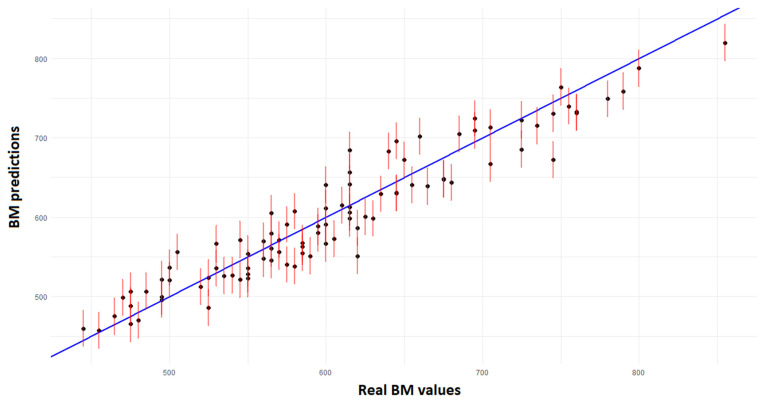
SVR model prediction graph.

The prediction graph of the SVR model is shown in Figure 11, which shows the relationship between the BM estimates and the significantly observed real values. The test results indicate a coefficient of determination (R^2^) of 0.9046, suggesting that the model explains a significant proportion of variability in the data. However, the obtained MAE and RMSE errors, with values of 22.81 kg and 27.41 kg, respectively, reveal that there is a margin of error in the predictions. These errors may impact the confidence in the estimates, particularly in practical applications in animal husbandry, where precision is critical for decision-making. Thus, despite the robust performance of the model, the analysis of errors suggests room for improvement, possibly through the inclusion of additional variables or adjustments to the modeling methods, as explored by [44].

### 3.7. Comparison Between Methods

A summary of the results of all models tested in this study is presented in Table 10.

The results obtained for the different BM prediction models for cattle show a significant variation in the performance of the approaches used. Table 10 presents the model results, including metrics such as the R^2^, MAE, and RMSE. The neural network model stood out with an R^2^ of 0.9139 on the training set and 0.9125 on the test set, in addition to the lowest MAE (21.97 kg) and RMSE (25.86 kg) values. These results highlight the neural network’s ability to capture complex relationships between variables and generate more accurate estimates.

The multiple models, specifically Multiple Model 1 (CP + AP + RW) and Multiple Model 2 (all variables), also demonstrated robust performance, with R^2^ values exceeding 0.89 and low relative errors (MAE around 22 kg and RMSE around 28 kg). This indicates that combining multiple variables is effective in improving the prediction accuracy. On the other hand, models using single variables, such as the CP model and the AP model, while simpler, proved to be viable alternatives in situations where the speed and ease of obtaining estimates are priorities.

Additionally, the SVR model results were promising, with an R^2^ of 0.9159 for the training set and 0.9046 for the test set. The SVR demonstrated an MAE of 22.81 kg and an RMSE of 27.41 kg, indicating a good fit. These results emphasize the effectiveness of SVR in modeling the relationship between morphological variables and cattle BM, showing competitiveness compared to other approaches, such as neural networks, which exhibited similar prediction behavior.

These results reinforce the idea that, despite the complexity of neural network and SVR techniques, incorporating different configurations and variables can potentially improve the model fit and estimation accuracy. The study in [7] utilized an advanced digital imaging system with the Microsoft Kinect device to estimate the body weight and carcass composition of cattle, achieving determination coefficients (R^2^) between 0.69 and 0.84, demonstrating the effectiveness of image-based methods. Similarly, the authors of [6] investigated the prediction of body weight in Girolando cattle using measurements extracted from images, highlighting the hip width and back area as highly correlated variables, with an R^2^ of up to 0.91. The authors in [11] identified a strong correlation between the thoracic circumference and live weight of cattle, with a correlation coefficient of r = 0.84, validating the importance of morphological measurements as reliable predictors. These studies show that while advanced techniques offer significant improvements, traditional methods combined with a robust dataset can also result in high accuracy for weight prediction.

In summary, the choice of the ideal model should consider the specific needs of the application scenario, balancing the precision of the estimates with simplicity and speed in obtaining results. Therefore, it is essential to recognize the practicality and feasibility of single-variable models, such as CP or RW, which, despite showing a poorer performance, still offer an efficient and accessible approach in certain field situations. For example, [39,45] exclusively used a single variable (GP—girth perimeter) as the predictor in their studies. The choice of this variable is due to its ease of measurement and its strong correlation with the BM of the animals, making it a reliable and practical indicator across various management conditions. While models with multiple variables may provide greater accuracy, using a single variable like CP simplifies the data collection process and can be particularly advantageous in contexts where simplicity and quick estimates are prioritized, as was likely the case for these authors.

Moreover, ensemble-based algorithms, such as Random Forest, represent a promising alternative to enhance the accuracy of BM estimation. The Random Forest Regressor stands out by combining multiple decision trees, reducing the risk of overfitting and improving the model’s generalization [46]. Its ability to capture non-linear relationships between variables can be advantageous in scenarios where multiple predictors are used, as in studies exploring complex morphological features to estimate BM [47]. Thus, future research could investigate the application of Random Forest for this task, comparing its performance with that of neural networks and other statistical models to assess its potential in different management contexts.

This scenario highlights the importance of integrating innovative and interdisciplinary approaches to address emerging challenges in digital and precision livestock farming, thereby expanding the reach and impact of research in the agricultural sector.

## 4. Conclusions

The results achieved in this study demonstrated that the development of predictive models based on machine learning techniques and morphometric variables of the back and sides of dairy cows was effective in accurately estimating the BM of the animals. Among the models tested, more complex approaches, such as neural networks and the SVR model, showed the best performances, with R^2^ values above 0.90 and low absolute errors (MAE of 21.97 and 22.81 kg, respectively), standing out as robust methods for BM prediction.

The comparison with simpler models, such as those based on single variables (CP, AP, and RW), revealed that although simplified approaches had lower R^2^ values of 0.7763, 0.7656, and 0.5484 with the test data, respectively, and higher mean error, they still offer a practical solution for scenarios where detailed data collection is not feasible. Moreover, the use of multiple-variable models, combining distinct variables such as the most significant ones (CP, AP, and RW), provided a significant improvement in accuracy, with an R^2^ of 0.9063 on the test data. This confirms that combining multiple morphometric parameters is advantageous for more precise BM prediction.

Therefore, the objective of developing models that integrate the physical dimensions of the animals was achieved. The proposed techniques, such as the use of neural networks and SVR, provide promising tools for the continuous monitoring of herds and the efficient management of animal health and welfare, contributing to more sustainable practices and the development of precision livestock farming.

The results obtained in this study demonstrate that the use of morphological measurements, even when collected manually, allowed for the construction of highly accurate predictive models for estimating cattle body mass. This method proves to be relevant for various applications, especially in scenarios where the direct collection of physical parameters is still the most viable and reliable approach. However, integrating these techniques with potential computer vision-based approaches can further enhance the efficiency of the process, reducing the need for manual measurements and facilitating automated data collection in large-scale production systems. Additionally, technologies such as passage scales can complement these strategies, making herd management even more efficient. Thus, the advances presented in this work represent a crucial step toward the development of hybrid solutions that combine morphological measurements and automated methods to improve the accuracy and applicability of precision livestock farming.

## Figures and Tables

**Table 1:** KS test results.

Variable	D¹	*p*-Value^2^	Conclusion
BM	0.071943	0.01624	Not Normal (*p* < 0.05)
CP	0.055219	0.1173	Normal (*p* > 0.05)
AP	0.054523	0.126	Normal (*p* > 0.05)
TW	0.06787	0.02758	Not Normal (*p* < 0.05)
AW	0.040716	0.4238	Normal (*p* > 0.05)
RW	0.081225	0.004329	Not Normal (*p* < 0.05)
DL	0.052554	0.1532	Normal (*p* > 0.05)
HH	0.052619	0.1523	Normal (*p* > 0.05)
BD	0.061925	0.05652	Not Normal (*p* < 0.05)

D^1^: The D (distance) statistic value from the KS test, measuring the maximum difference between the empirical cumulative distribution of the data and the theoretical cumulative distribution (normal, in this case). *p*-value^2^: The *p*-value associated with the test. If the *p*-value is less than 0.05, the null hypothesis that the data follow a normal distribution is rejected.

**Table 2:** Performance metrics of fitted models.

Model	Train R^2^	Test		
		R^2^	MAE	RMSE
Model CP	0.7953	0.7763	33.42	43.69
Model AP	0.7682	0.7656	34.23	44.72
Model RW	0.6062	0.5484	48.04	62.08

Here, CP = chest perimeter; AP = abdominal perimeter; RW = rump width; MAE = mean absolute error, and RMSE = root mean squared error are the performance metrics of regression models.

**Table 3:** Estimated coefficients.

Variable	Estimate	*p*-Value	Significance Level
CP	2.8999	<2 × 10^−16^	<0.001
AP	2.6445	<2 × 10^−16^	<0.001
RW	6.3471	<2 × 10^−16^	<0.001

**Table 4:** Performance metrics.

Model	Train R^2^	Test		
		R^2^	MAE	RMSE
Multiple Model 1 (CP + AP + RW)	0.8959	0.9067	22.29	28.00

**Table 5:** Estimated coefficients.

Variable	Estimate	*p*-Value	Significance Level
CP	2.7509	<2 × 10^−16^	<0.001
AP	2.3619	<2 × 10^−16^	<0.001
TW	0.8203	0.0636	Not significant
AW	0.6406	0.1471	<0.1
RW	5.6785	<2 × 10^−16^	<0.001
DL	0.3929	0.2054	<0.1
HH	−0.8360	0.0222	<0.05
BD	0.7616	0.1424	<0.1

Here, CP = chest perimeter; AP = abdominal perimeter; TW = thorax width; AW = abdomen width; RW = rump width; DL = dorsal length; HH = hip height; and BD = body depth.

**Table 6:** Performance metrics.

Model	Train R^2^	Test		
		R^2^	MAE	RMSE
Multiple Model 2 (All Variables)	0.8997	0.9063	22.20	28.05

**Table 7:** Hyperparameters chosen for the regression model.

Type of Optimizer	Number of Epochs	Topology	Batch Size
RMSprop	5000	3-10-20-10-1	8

**Table 8:** Performance metrics of the MLP model.

**Model**	**Train** **R^2^**	**Test**		
		R^2^	MAE	RMSE
ANN MLP	0.9139	0.9125	21.97	25.86

**Table 9:** Results obtained for the SVR.

Model	Train R^2^	Test		
		R^2^	MAE	RMSE
SVR	0.9160	0.9046	22.81	27.41

**Table 10:** Summary of results.

Model	Train R^2^	Test		
		R^2^	MAE	RMSE
Model CP	0.7953	0.7763	33.42	43.69
Model AP	0.7682	0.7656	34.23	44.72
Model RW	0.6062	0.5484	48.04	62.08
Multiple Model 1 (CP + AP + RW)	0.8959	0.9067	22.29	28.00
Multiple Model 2 (All Variables)	0.8997	0.9063	22.20	28.05
Artificial Neural Network	0.9139	0.9125	21.97	25.86
SVR Model	0.9159	0.9046	22.81	27.41

## Data Availability

The data collection process is detailed in the manuscript, and the range of collected values is also presented. The original contributions of this study are included in the manuscript, and further inquiries can be directed to the corresponding author.
